# Annotations

**Published:** 1905-09-30

**Authors:** 


### ANNOTATIONS.

### Hospitals and Ducal Residences.

Our contemporary, the *Sanitary Record*, calls attention in a leading article to the luxurious accommodation which is provided for the patients in county asylums at the present time. "It may be safely said," according to this article, "that no ducal residence in the country is better or more expensively constructed than the lunatic asylums which have been erected in recent years." Unfortunately there is a large measure of truth in this comparison; but the complaint need not be confined to lunatic asylums. The same ruthless extravagance is apparent in many, perhaps the majority, of our voluntary hospitals, as we have repeatedly pointed out; and the evil is a growing one. It is probably not far from the truth to say that the expenditure on building a modern hospital is twice what it ought to be. We should be the last to countenance any curtailment of the sick man's comforts, but economy in construction would not entail this in the very slightest degree. Cheaper materials could be used and all unnecessary architectural adornments omitted without any sacrifice of efficiency. It is true that a beautiful building is a thing to be desired; but the healing of the sick is yet more beautiful and desirable. When there is enough money and to spare for this purpose, then, and not till then, is it justifiable to indulge in expensive architectural ambitions. It is not a very good testimonial to the intelligence of the British public that hitherto they have overlooked this manifest principle. But we hope that they will awake soon and put an end to a cause of waste which is not creditable. As we have remarked before, matters are not likely to show much improvement until the arrangements with regard to the remuneration of hospital architects have been reconsidered. At present, the more a building costs, by so much the more in proportion is the architect's fee; and it does not need much worldly wisdom to perceive in which direction such a fact will tend.

### Motor Cars and Medical Practitioners.

There are probably but few people, or classes of people, who, when motor cars cease to be regarded as a luxury for the comparatively rich and come into common use as general methods of conveyance, will be more likely to make large use of them than medical practitioners. It is probably for this reason that the Joint Committee of the Automobile Club and the Motor Union of Great Britain and Ireland have selected the members of the medical profession as the recipients of a special circular, by the aid of which they are making preparations for encountering in Parliament the proposals of those who may desire to impose restrictions upon the use of cars, or penalties for the abuse of them, in excess of those which are already in force. Their object is to bring together a mass of evidence showing that, even now, motor cars are very largely employed for professional and other useful purposes; and further, that the offences charged against their drivers are insignificant when compared with their growing usefulness. We hope that this design may be successfully accomplished; for there can be no doubt that the objections to motors will die away as soon as they come into recognised use as the ordinary means of conducting highway traffic. A carriage and pair possesses enormous powers as an instrument of annoyance in the hands of anyone who desires to annoy; and, if it were placed on the roads now for the first time, its presence would no doubt be productive of columns of complaints in the newspapers. It is only because everyone understands its management, and that nobody turns round to look at it, that it is now taken as a matter of course. There can be no doubt that the motor car will soon attain the same privileged or recognised position, that the steering of its drivers will become orderly, and that its capabilities of speed will only be put forth on occasions which will justify their employment. If medical men can hasten the arrival of this good time by placing facts in the hands of the committee, they will by so doing be rendering a service both to their own profession and to the public.

### Viewing Bodies at Inquests.

At an inquest held on Monday at the Birmingham Coroner's Court, a protest was entered against the requirement that juries should view the body. The foreman of the jury, speaking on behalf of his fellow jurymen, begged, at the opening of the inquiry, that they might be excused from performing this unpleasant task. In reply, the Coroner is reported to have said that he thought it was absolutely outrageous that gentlemen should be called upon to see bodies such as he had seen; it was a terrible thing to have been called upon to do. He entirely agreed with the foreman of the jury, but they would have to walk to the hospital. He could not make them view the body, but they would go and walk past it. Although the protest is deserving of sympathy, the reasons attributed to the Coroner cannot be regarded as in any way satisfactory. For the circumstance that a public duty is unpleasant is not likely to be accepted by true citizens as a reasonable or proper ground for exemption; and, indeed, we do not see how a jury can reasonably be expected to decide upon the cause of death in a particular instance when they have not even seen the dead body. The fact is the system upon which inquests are conducted at the present day is antiquated, inefficient and cumbersome; it is every bit as out of date as the old jury of matrons. The principles of our legal system would not be violated if coroners' juries were abolished altogether, for an inquest is merely an inquiry, and in no sense a judicial tribunal. It is not to be supposed that twelve average laymen can appreciate the value of the medical evidence which is laid before them, and which is by far the most important evidence bearing on the cause of death in nearly all cases where inquests are held. The fact that they cannot gain any useful knowledge by inspection of the body is sufficient reason in itself for refusing to place any responsibility in their hands. Alternative methods are not difficult to suggest and have been seriously proposed more than once in the past. The face of the waters is troubled again now, and we trust that the outcome on this occasion will be a thorough overhauling of the entire system of death certification and of inquiry in all instances of unnatural or preventible death.

